# Oxidative Dehydrogenation of Propane over Vanadium-Containing Faujasite Zeolite

**DOI:** 10.3390/molecules25081961

**Published:** 2020-04-23

**Authors:** Małgorzata Smoliło, Katarzyna Samson, Ting Zhou, Dorota Duraczyńska, Małgorzata Ruggiero-Mikołajczyk, Agnieszka Drzewiecka-Matuszek, Dorota Rutkowska-Zbik

**Affiliations:** 1 Jerzy Haber Institute of Catalysis and Surface Chemistry, Polish Academy of Sciences, Niezapominajek 8, 30-239 Krakow, Poland; ncsmolil@cyf-kr.edu.pl (M.S.); ncsamson@cyf-kr.edu.pl (K.S.); tingzhou1995@yahoo.com (T.Z.); ncduracz@cyf-kr.edu.pl (D.D.); nbruggie@cyf-kr.edu.pl (M.R.-M.); ncmatusz@cyf-kr.edu.pl (A.D.-M.); 2 Faculty of Chemical Engineering and Technology, Cracow University of Technology, Warszawska 24, 30-155 Krakow, Poland

**Keywords:** oxidative dehydrogenation, catalysis, faujasite, zeolite Y, hierarchical zeolites, vanadium, propane

## Abstract

Oxidative dehydrogenation (ODH) of light alkanes to olefins—in particular, using vanadium-based catalysts—is a promising alternative to the dehydrogenation process. Here, we investigate how the activity of the vanadium phase in ODH is related to its dispersion in porous matrices. An attempt was made to synthesize catalysts in which vanadium was deposited on a microporous faujasite zeolite (FAU) with the hierarchical (desilicated) FAU as supports. These yielded different catalysts with varying amounts and types of vanadium phase and the porosity of the support. The phase composition of the catalysts was confirmed by X-ray diffraction (XRD); low temperature nitrogen sorption experiments resulted in their surface area and pore volumes, and reducibility was measured with a temperature-programmed reduction with a hydrogen (H_2_-TPR) method. The character of vanadium was studied by UV-VIS spectroscopy. The obtained samples were subjected to catalytic tests in the oxidative dehydrogenation of propane in a fixed-bed gas flow reactor with a gas chromatograph to detect subtract and reaction products at a temperature range from 400–500 °C, with varying contact times. The sample containing 6 wt% of vanadium deposited on the desilicated FAU appeared the most active. The activity was ascribed to the presence of the dispersed vanadium ions in the tetragonal coordination environment and support mesoporosity.

## 1. Introduction

As a basic raw material, low-carbon olefins such as ethylene and propylene play an important role in the petrochemical industry. Ethane and propane are the most important lower alkanes and are widely used in the industry. Ethane is mainly derived from the gaseous product of the cracking gas of the ethylene production unit, while propane is mainly obtained from the gaseous products of the delayed coking and catalytic cracking unit. To efficiently convert low-carbon alkanes to olefins, a dehydrogenation (DH) catalyst is required. At present, there are mainly two types of catalysts for the industrial dehydrogenation of lower alkanes to olefins: Pt-based catalysts and Cr-based catalysts [1]. Although they are widely used, they exhibit also certain disadvantages. Pt-based catalysts are expensive, and the active components are prone to sintering during the reaction process. In addition, the quality of raw materials should be high. Cr-based catalysts are highly toxic, harmful to human health and the environment, have poor stability, and are short-living, which leads to their loss. Therefore, there is a need for a new catalyst with high activity, selectivity and stability, low cost, and low environmental pollution able to efficiently convert low-carbon alkanes into olefins. Oxidative dehydrogenation (ODH) of light alkanes:

C_n_H_2n+2_ + 0.5 O_2_ → C_n_H_2n_ + H_2_O

is a promising alternative to the dehydrogenation process. The energy demand for this reaction is much lower than for dehydrogenation processes, as well as conventional catalytic cracking [2]. Other advantages of the ODH reaction include its exothermicity, lower operation temperatures, and minimization of the carbon deposit due to the oxidizing conditions [3]. The main shortcoming of the ODH process, however, is a parallel deep oxidation of the substrates and products [4,5,6,7,8,9]. There are also secondary problems, such as the removal of the reaction heat, flammability of the reaction mixture, and the possibility of the reaction runaway.

The literature reviews the current status of the research in the selective oxidation of light hydrocarbons [2,4,5,6,7,8,9]. The commonly used catalysts for the oxidative dehydrogenation of lower alkanes include vanadium-based systems [8,9,10], V-Mg-O systems [11,12,13], molybdate systems [14,15], rare earth metal systems [8], phosphate systems [16,17], and boron nitride systems [18]. An important factor in the design of efficient catalysts for alkane ODH is the isolation of the active sites [2,19]. This principle was formulated first by Callahan and Grasselli [20] and states that it is desired to provide enough active oxygen atoms at the isolated side to satisfy the stoichiometric requirement for the oxidation of the hydrocarbon to the desired product or to an isolable intermediate but less oxygen than is required for complete oxidation to waste products. There are different strategies to achieve active site isolation, such as the intermediate oxidation state, selection of metal oxides with naturally grouped active oxygen atoms in a field of less active oxygen atoms, and modification of an oxide surface by less than stoichiometric reaction with a reagent, which permanently renders a portion of it inactive [6,20].

Zeolitic materials constitute the type of supports which are particularly suited to achieve isolation of the active sites. Their advantages include large specific surface area, porosity, and thermal resistance, which favor their use in the chemical industry. Microporous materials may serve as support for vanadium, being promising ODH catalysts [21,22]. Different zeolite-based materials were tested in the oxidative dehydrogenation of alkanes. Propane ODH was probed on the zeolites with the faujasite structure: sodium form of zeolite Y and ultra-stabilized Y (USY) modified with Ca, Mg, Sn, and Sb cations by ion-exchange, as well as impregnated with boron, gallium, and indium oxides [22]. Propane conversion did not exceed 14% for any of the tested catalysts in the temperature range between 400–520 °C. The selectivity to propene (at 10% propane conversion) was 13.3% and 14.7% for Ca-Y and Mg-Y, respectively. The samples based on ion-exchanged USY exhibited higher selectivity at 10% propane conversion, ranging from 14.8% for In-USY up to 30.8% for Ca-USY. ZSM-5 zeolite with Ga was also tested, and its selectivity to propene was the highest and equaled 40.4% at a 10% C_3_H_8_ conversion. The authors concluded that the selectivity towards propene increases with the increase of the Si/Al ratio.

Materials containing vanadium introduced into the zeolite matrix were also used as catalysts for the oxidative dehydrogenation process [21,23]. The series of catalysts in which vanadium were introduced into modified BEA zeolite of the Si/Al ratio is equal to 1300 exhibited propane conversions lower than 14%. The selectivity to propene reached up to 63% but at C_3_H_8_ conversions not exceeding 2%. Similarly, vanadium-containing silicalites were also used for propane oxidative dehydrogenation with the C_3_H_8_ conversion up to 30% [24]. The highest selectivity to C_3_H_6_ (75%) was detected at 4% C_3_H_8_ conversion. Catalysts composed of vanadium introduced into MCM-22 exhibited propane conversions up to 20% [25]. The highest selectivity was 36.2% at the C_3_H_8_ conversion ca. 2%. Authors claimed that the mild acid sites collaborate with vanadium sites to promote higher selectivities towards propene. The comparison of activity of MFI-based membrane reactors for C_3_H_8_ ODH showed that V-aluminosilicates were more active than aluminosilicates in terms of propane conversion, which was equal to 19.5% and 14.1%, respectively, with the selectivity to propene equal to 40.6% in both cases [26]. To our knowledge, vanadium-loaded faujasite was not tested in the propane ODH process.

The aim of the present work was to synthetize vanadium containing zeolite systems using different forms of the faujasite zeolite (FAU) as a support to be tested as catalysts in the oxidative dehydrogenation of propane. The samples differed by the vanadium content and the type of FAU used as support. The vanadium was introduced via the classical wet impregnation method, and its content varied from 1.0 to 6.0 wt%. The faujasite (FAU) zeolite was selected as an example of the highly ordered microporous support. Additionally, a desilicated faujasite (FAUdes) was also used to check how the creation of additional mesoporosity influences the properties of the catalyst. The typical FAU zeolite has a low Si/Al ratio of typically 6 ± 1. Kubacka and co-authors concluded that the selectivity towards propene increases in line with the increase of the Si/Al ratio [22]; therefore, we decided to use the zeolites of the FAU structure in which the Si/Al was increased (to 31 and 18 in the FAU and FAUdes series, respectively) to reach a higher selectivity to propene. By selecting vanadium as the active phase of the catalyst, we wanted to shed light on to the factors responsible for the catalytic properties of vanadium catalysts, which are not entirely clear despite the intensive studies during last decades [3]. There is an ongoing debate on the type of vanadium centers involved in the oxidative dehydrogenation of alkanes. While some claim that the highest catalytic activity should be attributed to the isolated, single vanadium centers, some argue that the activity should be linked to the polymeric V-O-V chains or, perhaps, vanadium alone in tetrahedral or octahedral coordination.

## 2. Results and Discussion

Two series of the vanadium systems, V_x_FAU and V_x_FAUdes, in which faujasite and desilicated faujasite, respectively, were used as the support. In the following the structure, morphology, physico-chemical characterization, and catalytic activity of the obtained samples are discussed.

### 2.1. Structure and Morphology of the Obtained Samples

**Figure 1:**
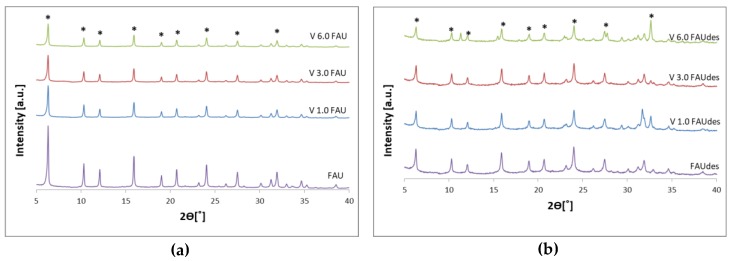
X-ray diffraction (XRD) diagrams of the obtained V_x_FAU (**a**) and V_x_ desilicated FAU (FAUdes) (**b**) samples. Peaks corresponding to the faujasite (FAU) crystal phase are marked with asterisks (*).

The phase composition and crystallographic structure of the samples was examined by X-ray diffraction (XRD) analysis to check the extent to which the crystal structure of the samples was preserved after the introduction of vanadium ions and/or the treatment with desilicating agents (TBAOH and NaOH). Figure 1 presents the obtained diffractograms.

All samples have a crystalline structure. The XRD patterns of the V_x_FAU vanadium catalysts and the parent FAU zeolite are similar, and no diffraction peaks due to crystalline phases other than the FAU structure are observed but for the V_6.0_FAUdes sample, for which an additional peak at 2Ø = 11° can be observed. It was attributed to the ammonium vanadium oxide (ammonium vanadate). The obtained diffractograms suggest that the zeolitic support keeps its crystalline structure, and the vanadium ions are well dispersed in the FAU matrix. In the FAUdes support, the characteristic peaks of the FAU zeolite structure can be seen, indicating the parent zeolitic structure is retained despite desilication process. These characteristic peaks are further visible for all V_x_FAUdes catalysts, which proves that the structure of the support has not changed. The FAUdes support exhibits less crystallinity compared to FAU, in agreement with earlier reports [27,28]. The impregnation with vanadium does not affect the support structure. The vanadium phase is well-dispersed; in all samples but V_6.0_FAUdes, it forms crystals small enough to be below the XRD detection limit or the vanadium phase is amorphous.

**Figure 2:**
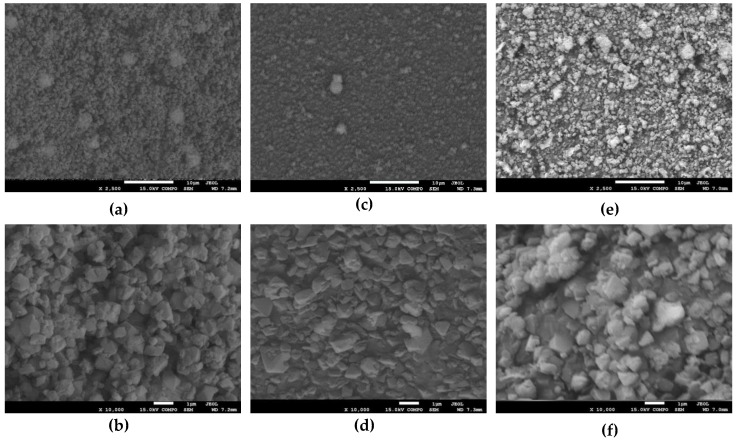
Scanning electron microscopy (SEM) images of the prepared samples: FAUdes (**a**,**b**), V_1.0_FAUdes (**c**,**d**), and V_3.0_FAU (**e**,**f**).

The morphology of the prepared catalysts was studied by using the scanning electron microscopy (SEM) technique—see Figure 2 for the exemplary SEM images.

The samples are composed of small, uniform grains of ca. 500 nm. In order to check the distribution of vanadium, the x-ray fluorescence spectroscopy was used (see Table 1), further supported with the energy-dispersive X-ray (EDS) spectroscopy results (see Table S1 in the Supplementary Information File). The vanadium loading obtained from both X-ray fluorescence (XRF) and EDS confirms the theoretical one within the experimental accuracy of the techniques. In the case of V_6.0_FAU, the actual vanadium load exceeds slightly the theoretically planned one.

Table 2 gathers data obtained from the porosimetric studies.

All materials have well-developed specific surface areas. The desilication of the FAU material resulted in the changes of zeolite porosity. The mesopore volume increased from 0.22 cm^3^/g (for FAU) to 0.63 cm^3^/g (for FAUdes), and the specific surface area related to mesoporosity raised from 158 m^3^/g (for FAU) to 423 m^3^/g (for FAUdes). These changes were at the cost of the microporosity and agree with previous literature data [27]. After the incorporation of vanadium atoms into the FAU zeolite, the specific surface area decreased. The higher the vanadium loading, the lower the specific surface areas and the lower the pore volumes are. The effect should be attributed to pore blocking due to the formation of vanadium oxide, which is probably formed at pore entrances. In the case of the VxFAUdes series, a drop of the specific surface area is observed only for the V_6.0_FAUdes sample. This may indicate a higher dispersion of the vanadium phase in this series of the catalysts.

### 2.2. Physico-Chemical Characterization of the Obtained Samples

**Figure 3:**
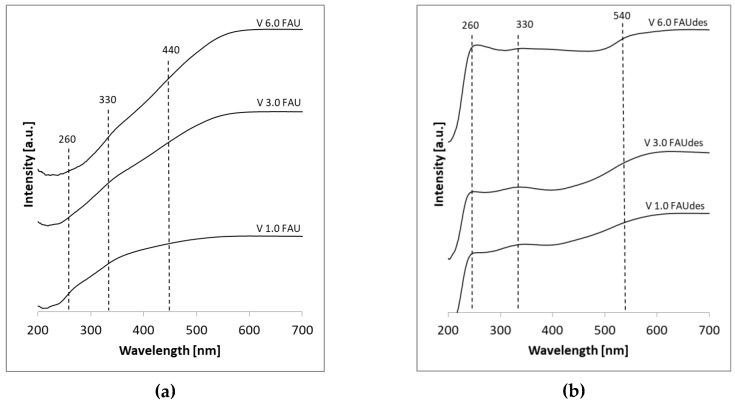
DR-UV-VIS spectra of the prepared V_x_FAU (**a**) and V_x_FAUdes (**b**) samples.

The diffuse reflectance UV-VIS spectra of the obtained samples were recorded (see Figure 3) to get insight into the nature and environment of the vanadium in the V_x_FAU and V_x_FAUdes series.

**Figure 4:**
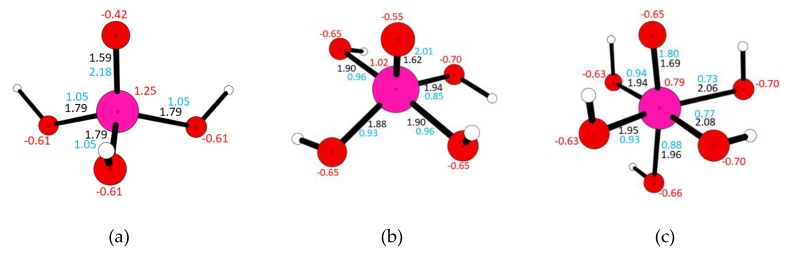
Geometry and electronic parameters of the VO(OH)_3_ (**a**), VO(OH)_4_^−^ (**b**), and VO(OH)_5_^2−^ (**c**) complexes.

The peak positions were compared with the characteristic peaks for different vanadium compounds, both derived from the literature data and resulting from the time-dependent density-functional theory (TDDFT) calculations performed for the model vanadium compounds—see Table 3 and Figures S1–S3 in the SI file. As model compounds, the VO(OH)_3_, VO(OH)_4_^−^, and VO(OH)_5_^2−^ complexes are chosen, as the vanadium ions have the analogous coordination of vanadium as in the common vanadium phases. The geometries of the model vanadium compounds, as well as their electronic parameters (bond orders and Mulliken charges), are depicted in Figure 4. As can be seen, the V=O (vanadyl) bond length ranges between 1.59 Å and 1.69 Å. The higher the coordination number, the longer the V=O bond. The character of the double-bond is confirmed by the bond order—that is, equal to 2.18–1.80. All V-OH bonds are longer (1.79 Å–2.08 Å) and of single-bond character (bond orders 0.73–1.05). Vanadium ions are positively charged, being the nucleophilic centers of the complexes. The highest charge is accumulated on the metal center of VO(OH)_3_ (q = 1.25), the lowest on the metal center of VO(OH)_5_^2−^ (q = 0.79). Out of the two types of oxygen atoms, those forming vanadyl groups are less charged (−0.42–−0.65) than those from hydroxyl groups (−0.61–−0.70).

In the case of the V_x_FAU series, the broad adsorption band for vanadium (V^5+^) ions is observed in the 300–500 nm range, evidencing the presence of V^5+^ ions of both tetra- and octahedral coordination. The former are found mostly in the V_1.0_FAU sample (and, to a lesser extent, in V_3.0_FAU), whereas octahedral vanadium species are present mostly at higher loadings, i.e., in the V_3.0_FAU and V_6.0_FAU samples. In the case of the V_x_FAUdes series, the maximum adsorption peak at 260 nm is present in the DR-UV-VIS spectra, which can be assigned to the presence of tetrahedral monomeric (isolated VO_4_) or tetrahedral 1D chains (polymerized VO_4_). The presence of the V^5+^ ions in the octahedral coordination can be deduced from the wide absorption peak above 500 nm.

The reducibility of the samples was assessed with temperature-programmed reduction with the hydrogen (TPR-H_2_) method. Both supports (FAU and FAUdes) are not reducible in the studied temperature range (results not shown).

**Figure 5:**
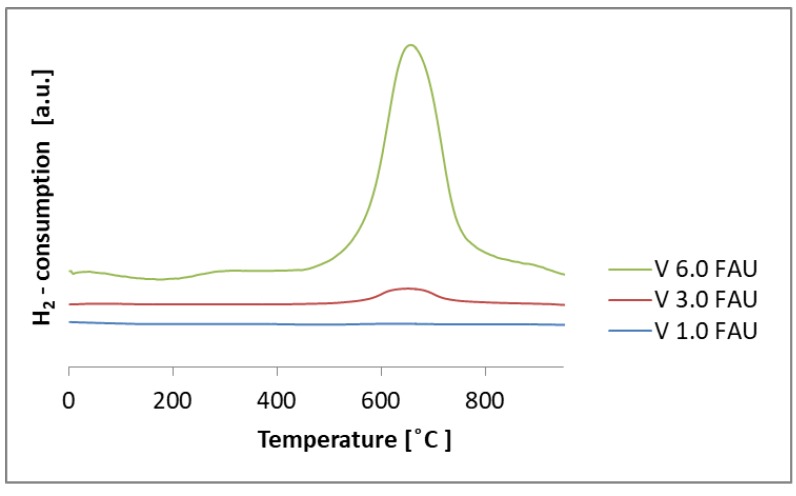
Temperature-programmed reduction with the hydrogen method (H_2_-TPR) profile of the prepared samples.

Figure 5 shows the reduction profiles of the V_x_FAU series.

It can be seen that, in V_1.0_FAU and V_3.0_FAU, there is only one reduction peak at 670 °C. This corresponds to the reduction of the vanadium species and suggests that the vanadium phase is of uniform character in these materials. In contrast, for V_6.0_FAU, besides the broad peak at 670 °C, a smaller one is observed at 840 °C. This may indicate the existence of yet another form of vanadium species that is present on the FAU support. TPR-H_2_ studies of pure or supported V_2_O_5_ catalysts reported in the literature show a single reduction peak at ~600 °C or multiple reduction peaks located at 461, 661, 698, and 860 °C. The presence of several peaks was ascribed to successive steps of the reduction of V_2_O_5_ to V_2_O_3_ via V_6_O_13_ and V_2_O_4_ or to the heterogeneity of the V-O centers, which are reduced during the experiment [29]. As the vanadium loading increases, the height of the peak also increases, because the hydrogen consumption corresponds to the number of vanadium-bound oxygen species on the surface.

Finally, the acidic properties of the studied catalysts were evaluated by performing NH_3_ temperature-programmed desorption experiments. The NH_3_ temperature-programmed desorption curves are presented in the Supplementary Information. The presence and location of peak maxima allow for the distinction of the acid centers of a given strength. Usually, the acid sites were classified into the very weak (<150 °C), connected with the physisorption of ammonia, weak (150–250 °C), medium (250–350 °C), and strong (350–500 °C). In order to relatively quantify the amount of acid sites of different strengths, the integration under each peak was done, and the desorbed amount of ammonia was computed—see Table 4. For the parent FAU system, two peaks are observed corresponding to the weak and medium acid centers, and the relative contribution of these sites is ca. 1:3. According to the Monte Carlo simulations for different zeolites of the faujasite structure [30], these correspond to ammonia interactions with the acid sites located in the sodalite units and supercages of the FAU support and is in line with the previous IR and solid-state NMR studies [28,31]. Impregnation of FAU with vanadium results in the lowering of the total acidity of the samples. This is due firstly to the exchange of acidic protons with vanadium ions. In V_1.0_FAU, the amount of weak acid sites is increased at a cost of the medium ones. The appearance of a low number of strong sites is also observed. When the amount of vanadium increases, medium acid sites reappear. The V_3.0_FAU catalyst is characterized by very low acidity (4.2 mmol NH_3_/g). The total acidity of V_6.0_FAU is comparable to that of the support (16.6 mmol NH_3_/g vs. 17.7 mmol NH_3_/g). Weak and medium acid sites are found on the surfaces of the V_x_FAUdes series. Out of those, the lowest acidity is measured for the V_1.0_FAUdes and V_6.0_FAUdes systems and is equal to 2.2 and 2.0 mmol NH_3_/g, respectively, while the total concentration of the acid sites in V_3.0_FAUdes (14.0 mmol NH_3_/g) is comparable to that of V_6.0_FAU. One should note that, in the V_x_FAU and V_x_FAUdes samples, a fraction of the acid sites is present on the vanadium phase: Lewis sites being unsaturated vanadyl groups and Bronsted sites associated with the V-OH groups. The sites that are located at the interface between the active phase and the zeolite support also contribute to the overall acidity. The literature shows that the acidity of the FAU and FAUdes supports was already studied with CO as the probe molecule. Although CO is a mild reducing agent, it is often used as a probe molecule to study the acidity of zeolites [28,32]. CO interacts with the acidic OH groups via its lone electron pair. As a result, the shift of the corresponding OH band, which is an indirect measurement of acid strength, is observed [32]. The experiments are done at a low temperature (room temperature or −100 °C), allowing only for the physisorption of CO and not the reduction of the surface. Our IR studies with CO suggest that both Lewis (v_C=O_ = 2200 cm^−1^), which may be either Al or V, and Bronsted acid sites (v_C=O_ = 2180 cm^−1^) are present after impregnation with vanadium. Exemplary spectra are shown in Figure S4 in the Supporting Information.

### 2.3. Determination of the Catalytic Properties in the Oxidative Dehydrogenation of Propane

**Figure 6:**
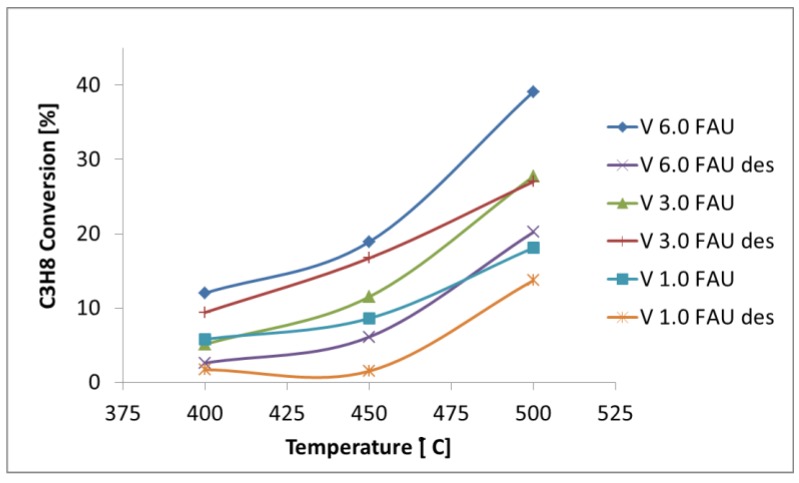
Conversion of propane as a function of the reaction temperature (contact time = 2 s).

**Figure 7:**
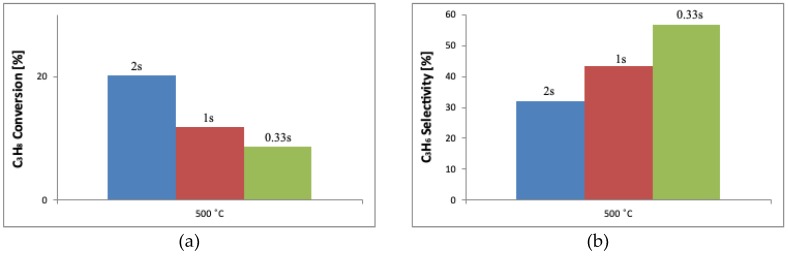
The conversion of propane (**a**) and selectivity to propene (**b**) as a function of the contact time (data plotted for the V_6.0_FAUdes catalyst for T = 500 °C).

**Figure 8:**
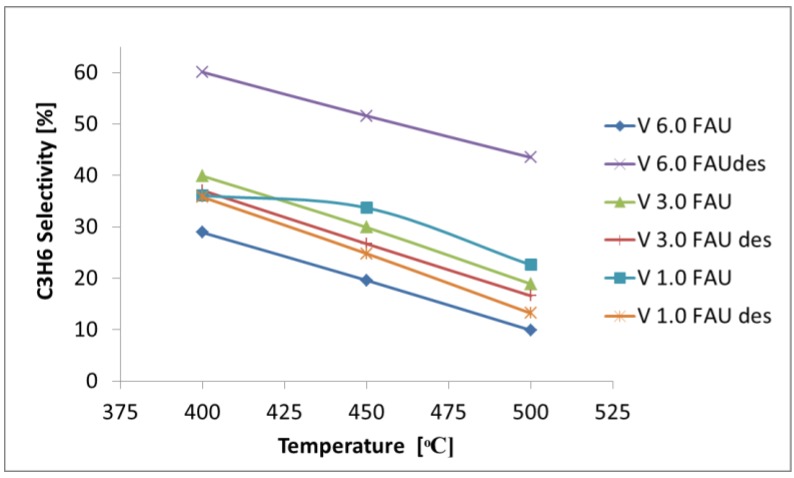
Selectivity to propene as a function of the reaction temperature.

**Figure 9:**
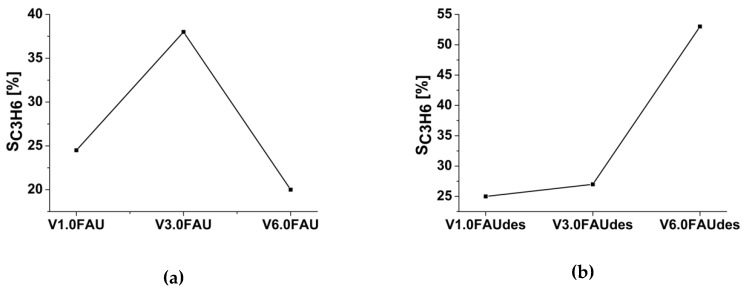
Selectivity to propene at a constant conversion equal to 10% (±2%) at 450 °C for the V_x_FAU (**a**) and at constant conversion equal to 5% at 450 °C for the V_x_FAUdes (**b**) series.

As the reaction products, propene (the desired product of the ODH process) and carbon oxides (CO and CO_2_ as products of the alkane deep oxidation) were detected. Ethylene formation was also monitored, but the selectivity to C_2_H_4_ never exceeded 3%. The results of all performed catalytic tests are gathered in Tables S2–S7 (in the Supplementary Information File) and presented in Figure 6, Figure 7, Figure 8 and Figure 9.

All catalysts proved active in the ODH of propane to propene. The higher the reaction temperatures, the higher the conversion of propane is observed (see Figure 6). The measured propane conversions are below 40%. Such rather low conversions are typical for the ODH process—see, e.g., [29,33].

Figure 7 shows the relation between the catalytic performance (C_3_H_8_ conversion and C_3_H_6_ selectivity) and the contact time, taking as the example the V_6.0_FAUdes sample. For the catalytic tests performed at the same temperature, usually the shorter the contact times are, the lower the propane conversion is. The selectivity to propene increases in line with the decrease of contact time. Such an observation suggests that the prolonged contact between the substrate and the catalyst leads to the deep oxidation of C_3_H_8_ rather than its selective oxidation. The nature of the catalyst may offer a possible explanation of the result. Here, the reaction probably takes place inside the pores of the zeolite rather than on its external surface. The inclusion of the reactant inside the zeolitic pores, close to the active phase and their prolonged contact, may lead to propane combustion. The effect can be further enhanced by the existence of acidic groups inside the zeolite channels, which can form carbocations, leading to the undesired hydrocarbon transformations.

Further, the examination of the catalytic data reveals that, in general, the propane conversions are higher for the V_x_FAU series than for the V_x_FAUdes series. This may result from the differences in the internal structure of the used zeolites and the additional mesoporosity of FAUdes in comparison to FAU. One may also try to correlate the activity of the catalysts with their acidity. Literature data suggests that lowering the number of strong Brønsted acid sites can modulate the cracking tendency of the catalysts [24], which, in turn, enhances its selectivity. Indeed, on the most active catalysts, there are not strong acid sites according to the temperature-programmed desorption of ammonia (NH_3_-TPD) results. The experiments showed that, in V_x_FAUdes, weak and medium acid sites are present. Further, the V_1.0_FAUdes and V_6.0_FAUdes are characterized by low overall acidity (see Table 4). The presence of the acid sites inside the pores of the zeolitic catalyst can be beneficial for ODH, as they serve as the “anchoring” sites for propane. On the other hand, they may promote the cracking of alkane, leading to its deep oxidation and loss of selectivity.

In the propane ODH, one is interested in obtaining propene in the highest possible yields. Therefore, the selectivity to C_3_H_6_ was selected as the main parameter to measure the catalytic performance of the obtained samples. Figure 8 presents how the propene selectivity depends on the reaction temperature for all the tested samples.

The higher reaction temperature, although being of favor of propane conversion, is accompanied by a lower selectivity to propene. At the same time, higher CO_2_ selectivities are observed, i.e., a higher reaction temperature promotes the deep oxidation of alkanes. Such a behavior is typical for the ODH reactions on oxide-based catalysts [34] and is due to the nonselective oxidation of hydrocarbons, leading to carbon dioxide. It should be noted that the selectivity to CO_2_ stays at the same level irrespectively of the contact time and is, rather, a function of the reaction temperature.

In the whole temperature range, the highest selectivity to propene is found for the V_6.0_FAUdes sample, while the lowest selectivity to propene is found for the V_6.0_FAU sample. The remaining samples exhibit selectivity comparable one to another, but their overall performance resembles that of the V_6.0_FAU catalyst. This behavior may be linked to an increased mesoporosity of the sample or the low acidity of the catalyst, as evidenced by NH_3_-TPR.

The direct comparison of the catalytic activity (i.e., the selectivity to C_3_H_6_) within each of the series can be done only at the constant conversion regimes. As the reference conditions, the conversion of 10% (±2%) at 450 °C was chosen for the V_x_FAU series—see Figure 9a. It seems that the best activity is achieved for the V_3.0_FAU sample. The selectivity to propene reached 37.5%. As can be noticed, the samples of both lower and higher vanadium loading exhibit a lower selectivity to propene. The V_3.0_FAU catalyst is characterized by the lowest total acidity from the series. For the V_x_FAUdes series, the conversion of 5% at 450 °C was chosen (see Figure 9b), as these samples exhibit lower conversions, and 10% conversion is difficult to acquire for all the V_x_FAUdes catalysts in this temperature. Here, the selectivity to propene increases with the increasing vanadium content. The best result is achieved for the V_6.0_FAUdes sample, for which a 51.6% selectivity to propene is measured.

The abovementioned observations can be explained by the character of the vanadium active phase, as seen by the UV-VIS technique. It seems that the samples in which vanadium is present in the tetrahedral coordination exhibit higher selectivity to propene than those in which vanadium is present in the octahedral coordination. Indeed, in the V_6.0_FAU sample, vanadium is located mostly in the octahedral environment, while, in the V_1.0_FAU and V_3.0_FAU catalysts, vanadium is located in the tetrahedral positions. As deduced from Figure 3, in all the V_x_FAUdes samples, one finds both tetrahedral and octahedral vanadium ions. We are aware that our investigations are not exhaustive, and further studies are needed in order to elucidate the nature of the active vanadium phase. This would include the studies of vanadium speciation, possibly with the x-ray absorption (XAS) technique and IR studies. The same location of the vanadium active phase in the samples can be investigated in more detail. At the moment, we are not able to distinguish between the V species distributed inside the pores and those at the outer surfaces in our samples.

We have chosen the V_3.0_FAU and V_3.0_FAUdes samples to compare both series of the catalysts. While containing the same amount of the vanadium active phase, they differ by the properties of the support, mostly by its mesoporosity. To compare the samples, the selectivity to propene at a 10% conversion at 450 °C was chosen. The selectivity to propene is slightly higher for V_3.0_FAUdes (41.2%) than for V_3.0_FAU (37.5%). The difference is not substantial, but it suggests that the increased porosity of the FAUdes samples facilitates propene synthesis and, thus, impacts the catalytic activity of the systems.

Finally, the stability tests of the tested catalysts were done. The activity of the sample after 40 h on stream was measured at 450 °C and compared with the activity of the same sample at 450 °C after one hour on stream—see Table S8 in the Supplementary Information File. Both the propane conversions and the propene selectivities are comparable, and only a minor loss of activity was observed. This may indicate that, after 40 h of the reaction, only part of the catalyst became deactivated.

## 3. Materials and Methods

### 3.1. Catalyst Preparation

Two series of vanadium catalysts with varying vanadium contents (1wt%, 3 wt%, and 6 wt%) were prepared. The catalysts were prepared by classical wet impregnation using a water solution of ammonium metavanadate (NH_4_VO_3_), supplied by POCh, Avantor Performance Materials Poland S.A., Gliwice, Poland) as the source of vanadium and two forms of the faujasite zeolite as support (see below). The pH of the solution was stabilized at 2.5. The suspension was stirred constantly for 24 h at RT and evaporated under the vacuum for 2 h in air at 80 °C until water was removed. The obtained samples were calcined in air flow for 3 h at 500 °C.

For the first series, the protonic form of the faujasite-type zeolite of Si/Al = 31 was used as supplied by the Zeolyst International Company (CBV 760, Farmsum, The Netherlands). The Si/Al ratio was confirmed by XRF measurements. The zeolite was dealuminated by steaming and acid treatment. These samples are further denoted as V_1.0_FAU, V_3.0_FAU, and V_6.0_FAU. The second series was prepared using the desilicated FAU (FAUdes) as the support. The desilication of the starting FAU system proceeded according to the procedure described in detail in [27] using the 0.2-M mixture of tetrabutylammonium hydroxide (TBAOH) and NaOH, yielding the support of an Si/Al ratio of 18, as confirmed by the XRF results. These samples are further denoted as V_1.0_FAUdes, V_3.0_FAUdes, V_6.0_FAUdes.

### 3.2. X-ray Diffraction (XRD) Analysis

The diffraction patterns of supports and catalysts were carried out using X-ray powder diffractometry on an XRD X’Pert PRD powder diffractometer, PANalytical. XRD patterns were recorded using CuKα radiation (λ = 1.54178 Å). Measurements (at 40 kV and 30 mA) were performed in the 2θ range from 5 to 50° with an interpolated step size of 0.033° every 12 min. XRD patterns were recorded at room temperature. Diffraction patterns were assigned to a given compound using the Cambridge Crystallographic Data Centre (CCDC) resources.

### 3.3. Specific Surface Area and Porosity Studies

The specific surface area (S_SA_) of the used FAU zeolites and the prepared catalysts was determined with the multipoint Brunauer-Emmett-Teller (BET) analysis method using an Autosorb-1 Quantachrome flow apparatus, with nitrogen as an adsorbate, at −196 °C. Prior to the measurements, all samples were preheated and degassed under vacuum at 200 °C for 16 h. Micropore pore volume and micropore surface were determined with the t-plot method, while the mesopore pore volume and mesopore surface were determined with the Barret-Joyner-Halenda (BJH) method.

### 3.4. Determination of Surface Morphology and Composition by Scanning Electron Microscopy (SEM) with the Energy-Dispersive X-ray Spectroscopy (EDS) Method

High-magnification SEM images were recorded using a JEOL JSM-7500F field emission scanning electron microscope (SEM) equipped with the X-ray energy dispersive (EDS) system—INCA PentaFetx3. The secondary electron detector provided SEI images, and the back-scattered electron detector provided BSE (COMPO) micrographs. K575X Turbo Sputter Coater was used for coating the specimens with chromium (deposited film thickness—30 nm).

### 3.5. Determination of Elemental Composition by the X-ray Fluorescence (XRF) Method

XRF spectroscopy was used to determine the elemental composition, namely the wt.% of V, Si, and Al of the prepared samples. The measurements were performed using the EDX 3600H apparatus by Skyray Instrument equipped with a tungsten lamp of 9 kV and 40 kV voltage to determine the Si and Al, as well as V, respectively.

### 3.6. Determination of Vanadium Coordination by the Ultra-Violet Visible Spectroscopy (UV-VIS) Method

UV-VIS spectra were obtained at 20 °C in the range 200–800 nm with a Perkin Elmer Lambda 9 spectrometer equipped with a reflectance accessory and a sample holder containing 0.2 g solid powder BaSO_4_, which was used as a reference in the measurements.

### 3.7. Quantum Chemical Calculations

The geometry of the model vanadium complexes in which vanadium(V) ions exhibited different coordination environments (tetrahedral, octahedral, and square pyramid) was computed within the density functional theory (DFT) by using the Perdew-Burke-Ernzerhof (PBE) functional [35,36,37,38] and def2-TZVP basis sets [39,40]. The theoretical absorption spectra were obtained with a time-dependent density-functional theory (TDDFT) approach with PBE/def2-TZVP. Prior to the TDDFT calculations, the energy and density convergence thresholds were set to 10^−7^ Hartree. Forty lowest lying singlet excited states were calculated for each of the studied systems. The calculations were done with Turbomole 6.3 [41].

### 3.8. Determination of Reducibility by Temperature-Programmed Reduction (TPR-H_2_)

The TPR-H_2_ measurements were carried out on the Quantachrome Chembet 3000 apparatus in the temperature range of 25–800 °C. About 0.025 g of the sample was placed in a flow “U”-type quartz reactor. Before the reduction, each sample was degassed for 1 h in a helium stream at 100 °C to remove physically adsorbed water. After this time, the sample was cooled down to an ambient temperature and then reduced using a mixture of H_2_ (5 vol.%) + Ar, with a flow of 30 mL/min and a temperature ramp of 10 °C/min. The TPR profiles were recorded using a thermal-conductivity detector TCD (katharometer).

### 3.9. Determination of Acidity by the Temperature-Programmed Desorption of Ammonia (TPD-NH_3_)

The NH_3_ temperature-programmed desorption measurements were carried out in a quartz fixed-bed flow reactor connected online to the mass spectrometer (QMG 220 PRISMA PLUS), allowing for monitoring of the m/z = 16 signal. Prior to the TPD run, the 50 mg samples were activated in the He flow (40 mL/min) at 350 °C for 0.5 h. Next, the reactor was cooled down to 60 °C, and a 3%NH_3_/N_2_ mixture was introduced for 0.5 h. Then, the sample were flushed in the He flow (40 mL/min) to remove the physisorbed NH_3_ and to obtain a stable NH_3_ line.

### 3.10. Determination of Catalytic Properties in the ODH (Oxidative Dehydrogenation) of Propane

The activity of the catalysts in the oxidative dehydrogenation of propane was measured in a fixed-bed gas flow reactor in the temperature range of 400–520 °C. A stainless steel reactor (120 mm length and 13 mm diameter) in the oven was coupled directly by a set of manifold valves to a gas chromatograph, the thermocouple for the temperature measurements being placed coaxially in the catalyst bed. Analysis of substrates (propane, O_2_, and N_2_) and products (unreacted propane, propene, ethylene, and CO_x_) was performed by on-line gas chromatography using GC Agilent technologies 7890B with TCD and FID detectors equipped with three capillary columns for qualitative and quantitative measurements. The reaction mixture contained 7.1 vol% of propane in the synthetic air. The gas flows were regulated by mass flow regulators: ERG for propane and Aalborg for synthetic air. The catalysts grains of 0.63-1-mm diameters (about 0.5 mL) were used for the catalytic tests, diluted with acid-washed quartz beads of the same diameters (1:1) in order to avoid temperature and concentration gradients. Analysis of the products and unreacted alkane was started after 1 h of stabilization in the reaction mixture at the given temperature. The selectivity to the given reaction product *i* was calculated from the number of moles of the product *i* divided by the total number of moles of products in the product mixture using the general formula:

$$S_{i}\left[\%\right]=\frac{x_{i}^{-1}n_{i}}{\sum^{\;}x_{i}^{-1}n_{i}}\cdot 100$$

while the conversion as:

$$\mathrm{conv}\;\left[\%\right]=\frac{\sum^{\;}x_{i}^{-1}n_{i}}{\sum^{\;}x_{i}^{-1}n_{i}+n_{C3H8}\left(\mathrm{output}\right)}\cdot 100$$

where *x_i_*—stoichiometric coefficient of the reaction leading to the product *i*, *n_i_*—number of moles of the product *i*, and n_C3H8_ (output)—number of moles of propane at the output.

In order to compute the carbon balance (C_B_), the conversion was compared with the conversion calculated based on the total conversion of propane (conv_t_):

$${\mathrm{conv}}_{t}\left[\%\right]=\frac{n_{C3H8}\left(\mathrm{input}\right)-n_{C3H8}\left(\mathrm{output}\right)}{n_{C3H8}\left(\mathrm{input}\right)}\cdot 100$$

where n_C3H8_ is the number of moles of propane at the input.

$$C_{B}\left[\%\right]=\frac{\mathrm{conv}}{{\mathrm{conv}}_{t}}\cdot 100$$

Both quantities are close to one another, resulting in the carbon balance equal to 96%–100%.

Prior to the catalytic data acquisition, two blank tests were performed. The first one was done with an empty reactor, which is made of stainless steel and, therefore, may exhibit non-zero catalytic activity. The test was carried in the temperature range of 280–520 °C with a gas flow of 30 mL/min (contact time was equal to 2 s). In the whole range of temperatures, the conversion of propane was equal to zero. The second blank test was done with the reactor filled with 1-mL quartz beads in the temperature range of 280–500 °C with a gas flow of 30 mL/min. No conversion of propane was detected below 500 °C. At 500 °C, the propane conversion was measured to reach 3%.

The catalytic activity of the prepared samples was then tested in different temperatures (400, 450, and 500 °C) under different gas flow regimes (30, 60, and 90 mL/min) corresponding to different contact times (2, 1, and 0.33 s, respectively).

## 4. Conclusions

In summary, the activity of the reported catalysts is the interplay of three parameters: vanadium location, porosity of the samples, and their acidity. The higher selectivity of the V_x_FAUdes catalysts should be mainly ascribed to the presence of the dispersed vanadium ions in the tetragonal coordination environment, as seen by the performed UV-VIS spectroscopy measurements.

Another important aspect is the support porosity. As shown, the conversions of C_3_H_8_ are higher for the V_x_FAU series than for the V_x_FAUdes series. The microporosity prevails over the mesoporosity in the V_x_FAU systems. The narrow pores hinder the diffusion of reagents inside the zeolite, resulting in the increased probability of the catalytic reaction. On the other hand, the prolonged contact with the active sites may lead to the combustion of propane. This explains the overall higher selectivity to propene found for the V_x_FAUdes systems.

Finally, a low total acidity of the catalysts, as found for V_3.0_FAU and V_6.0_FAUdes, favors a high selectivity to C_3_H_6_. The presence of weak acid sites promotes hydrocarbon adsorption on the catalysts’ surfaces, while the lack of strong acid sites impedes propane cracking.

These factors contribute to the overall catalytic activity, but their relative importance being not determined yet calls for further studies.

## Figures and Tables

**Table 1:** Results of the X-ray fluorescence (XRF) analysis of the studied vanadium catalysts. FAU: faujasite and FAUdes: desilicated FAU.

Sample	V Content (wt.%)
V1.0 FAU	1.07
V3.0FAU	3.55
V6.0FAU	8.13
V1.0FAUdes	1.26
V3.0FAUdes	4.45
V6.0FAUdes	6.62

**Table 2:** N_2_ physisorption-derived parameters characterizing the obtained samples (S_SA_—specific surface area).

Sample	S_SA_ (m^2^/g)	Pore Volume (cm^3^/g)	Micropore Pore Volume (cm^3^/g)	Mesopore Pore Volume (cm^3^/g)	S_SA_ Micropores (m^2^/g)	S_SA_ Mesopores (m^2^/g)
FAU	883	0.52	0.30	0.22	724	158
V_1.0_ FAU	750	0.47	0.27	0.20	625	125
V_3.0_FAU	704	0.45	0.27	0.18	580	124
V_6.0_FAU	670	0.42	0.24	0.18	565	105
FAU_des_	688	0.75	0.12	0.63	265	423
V_1.0_FAU_des_	669	0.73	0.13	0.60	314	355
V_3.0_FAU_des_	669	0.83	0.07	0.76	151	518
V_6.0_FAU_des_	603	0.65	0.12	0.53	285	318

**Table 3:** Time-dependent density-functional theory (TDDFT) (PBE/def2-TZVP) absorption peak positions for model V compounds and the reference experimental UV-VIS peak positions for vanadium systems.

Nature of Species	Peak Position (nm)	Remarks	References
Tetrahedral monomeric (isolated VO_4_)	240–290 280 240 253, 294	Compound: Na_3_VO_4_ (ortho-vanadate)	[22] [23] [24] [25]
	215, 225, 250, 291	Compound: VO(OH)_3_ TD-DFT results	This work
Tetrahedral 1D chains (polymerized VO_4_)	270–290 280, 340 288, 363 281, 353	Compound: NH_4_VO_3_; NaVO_3_ (meta-vanadate)	[22] [26] [27] [28]
Square pyramidal	410	Compound: α-VPO_5_	[22]
	234, 284, 424	Compound: VO(OH)_4_^−^ TD-DFT results	This work
Octahedral multilayer (polymerized VO_5_/VO_6_)	470 330–500 480 330, 460 334, 481	Compound: V_2_O_5_	[22] [23] [24] [26] [29]
	218, 232, 263, 320, 393, 452	Compound: VO(OH)_5_^2−^ TD-DFT results	This work

**Table 4:** Distribution of acid sites and total acidity as of the temperature-programmed desorption of ammonia (NH_3_-TPD) experiments.

Sample	Weak (mmol NH_3_/g) 150–250 °C	Medium (mmol NH_3_/g) 250–350 °C	Strong (mmol NH_3_/g) 350–500 °C	V. Strong (mmol NH_3_/g) 500–700 °C	Total Acidity (mmol NH_3_/g)
FAU	4.4	13.3	0	0	17.7
V_1.0_FAU	8.6	0	3.0	0	11.6
V_3.0_FAU	0	4.2	0	0	4.2
V_6.0_FAU	1.9	14.7	0	0	16.6
V_1.0_FAUdes	0.6	1.6	0	0	2.2
V_3.0_FAUdes	4.7	9.3	0	0	14.0
V_6.0_FAUdes	1.2	0.8	0	0	2.0

## Supplementary Materials

The following are available online: Figure S1: Theoretical (TD-DFT: PBE/def2-TZVP) UV-VIS spectrum of the VO(OH)_3_ complex, in which vanadium is located in the tetrahedral environment. Figure S2: Theoretical (TD-DFT: PBE/def2-TZVP) UV-VIS spectrum of the VO(OH)_4_^−^ complex, in which vanadium is located in the square pyramid environment. Figure S3: Theoretical (TD-DFT: PBE/def2-TZVP) UV-VIS spectrum of the VO(OH)_5_^2−^ complex, in which vanadium is located in the octahedral environment. Figure S4: Exemplary IR spectra of the studied samples. Figure S5: NH_3_-TPD profiles for the V_x_FAU (a) and V_x_FAUdes (b) series. Table S1: Elemental composition of the selected catalyst samples obtained from the energy-dispersive X-ray spectroscopy. Table S2: Results of the catalytic tests for the V_1.0_FAU sample. Table S3: Results of the catalytic tests for the V_3.0_FAU sample. Table S4: Results of the catalytic tests for the V_6.0_FAU sample. Table S5: Results of the catalytic tests for the V_1.0_FAUdes sample. Table S6: Results of the catalytic tests for the V_3.0_FAUdes sample. Table S7: Results of the catalytic tests for the V_6.0_FAUdes sample. Table S8: Comparison of the initial catalytic performance of the studied samples with their activities after 40 h (T = 450 °C, flow = 60 mL/min).
