# Supplementary material for: Oxidative Dehydrogenation of Propane over Vanadium-Containing Faujasite Zeolite
Source: Molecules. 2020 Apr 23;25(8):1961. doi: 10.3390/molecules25081961 (PMC7221564; doi:10.3390/molecules25081961)
Supplement: Supplementary file 1 [file molecules-25-01961-s001.pdf]

molecules

Supplementary Information file for the manuscript:

Oxidative Dehydrogenation of Propane over Vanadium-Containing Faujasite Zeolite

Małgorzata Smoliło [1], Katarzyna Samson [1], Ting Zhou[1,2], Dorota Duraczyńska [1], Małgorzata Ruggiero [1], Agnieszka Drzewiecka-Matuszek[1] and Dorota Rutkowska-Zbik [1,]*

[1] Jerzy Haber Institute of Catalysis and Surface Chemistry, Polish Academy of Sciences, Niezapominajek 8, 30-239 Krakow, Poland; ncsmolil@cyf-kr.edu.pl; ncsamson@cyf-kr.edu.pl; ncduracz@cyf-kr.edu.pl; nbruggie@cyf-kr.edu.pl; ncmatusz@cyf-kr.edu.pl; nczbik@cyf-kr.edu.pl

[2] Faculty of Chemical Engineering and Technology, Cracow University of Technology, Warszawska 24, 30-155 Krakow, Poland; tingzhou1995@yahoo.com

* Correspondence: nczbik@cyf-kr.edu.pl; Tel.: +48-12-639-5160 (D. R.-Z.)

Table S1. Elemental composition of the selected catalyst samples obtained from the energy-dispersive X-ray spectroscopy.

Sample	O [wt%]	Al [wt%]	Si [wt%]	V [wt%]
$V_{1.0}$FAUdes	76.6	1.2	21.3	0.9
$V_{3.0}$FAUdes	67.4	0.8	27.1	2.4
$V_{5.0}$FAU	68.9	0.7	37.7	3.7

Table S2. Results of the catalytic tests for the $V_{1.0}$FAU sample.

SAMPLE	T_r [°C]	Flow [ml/min]	Conv [%]	S_{C3H6} [%]	S_{C2H4} [%]	S_{CO2} [%]	S_{CO} [%]
$V_{1.0}$FAU 0.5ml/0.5ml quartz/catalyst	400	30	5.8	34.7	0.8	43.8	20.7
		60	2.7	36.1	0.7	48.8	14.3
		90	4.4	49.1	0.9	36.4	13.6
	450	30	8.6	24.6	1.0	47.9	26.4
		60	7.7	33.7	1.0	42.9	22.5
		90	7.2	41.6	0.9	39.1	18.4
	500	30	18.1	14.4	3.0	50.7	31.8
		60	15.6	22.6	2.2	46.1	29.1
		90	15.6	22.6	2.2	46.2	29.1

Table S3. Results of the catalytic tests for the $V_{3.0}$FAU sample.

SAMPLE	T_r [°C]	Flow [ml/min]	Conv [%]	S_{C3H6} [%]	S_{C2H4} [%]	S_{CO2} [%]	S_{CO} [%]
$V_{3.0}$FAU 0.5ml/0.5ml quartz/catalyst	400	30	5.1	30.3	0.5	43.2	26.0
		60	21.7	39.9	0.5	38.4	21.1
		90	17.0	46.3	0.4	31.3	21.9
	450	30	11.5	22.0	0.9	47.4	29.8
		60	13.0	29.9	0.6	43.3	26.1

		90	8.8	37.5	0.5	35.6	26.4
	500	30	27.7	12.1	2.5	51.1	34.3
		60	26.3	18.9	1.8	46.8	32.4
		90	19.4	24.5	1.3	42.2	31.9

Table S4. Results of the catalytic tests for the $V_{6.0}$FAU sample.

SAMPLE	T_r [°C]	Flow [ml/min]	Conv [%]	S_{C3H6} [%]	S_{C2H4} [%]	S_{CO2} [%]	S_{CO} [%]
$V_{6.0}$FAU 0.5ml/0.5ml quartz/catalyst	400	30	12.0	16.7	0.3	59.6	23.4
		60	20.1	28.9	0.3	47.4	23.4
		90	8.0	26.3	0.2	54.4	19.1
	450	30	18.9	10.9	0.8	64.2	24.1
		60	14.7	19.6	0.4	54.8	23.2
		90	12.8	18.9	0.5	58.9	21.7
	500	30	39.1	41.7	1.9	36.9	19.6
		60	40.4	9.9	2.5	61.5	26.1
		90	30.9	13.1	2.5	58.8	26.1

Table S5. Results of the catalytic tests for the $V_{1.0}$FAUdes sample.

SAMPLE	T_r [°C]	Flow [ml/min]	Conv [%]	S_{C3H6} [%]	S_{C2H4} [%]	S_{CO2} [%]	S_{CO} [%]
$V_{1.0}$FAUdes 0.5ml/0.5ml quartz/catalyst	400	30	1.7	13.8	0.4	69.2	16.5
		60	4.4	35.8	0	50.5	13.6
		90	10.9	22.5	0	66.7	10.8
	450	30	1.5	13.8	0.5	64.6	21.1
		60	3.7	24.8	0.6	57.4	17.2
		90	3.4	31.0	0.5	57.7	10.8
	500	30	18.1	14.4	3.0	50.7	31.8
		60	15.6	22.6	2.2	46.1	29.1
		90	15.6	22.6	2.2	46.2	29.1

Table S6. Results of the catalytic tests for the $V_{3.0}$FAUdes sample.

SAMPLE	T_r [°C]	Flow [ml/min]	Conv [%]	S_{C3H6} [%]	S_{C2H4} [%]	S_{CO2} [%]	S_{CO} [%]
$V_{3.0}$FAUdes 0.5ml/0.5ml quartz/catalyst	400	30	9.4	28.2	0.4	42.3	29.1
		60	6.5	37.1	0.5	37.4	25.0
		90	14.1	34.1	0.2	36.6	29.0
	450	30	16.7	19.1	0.9	47	33.0
		60	5.7	26.7	0.6	41.6	31.1
		90	10.2	41.2	0.9	39.6	57.3

		30	27.0	11.2	2.3	49.4	37.1
	500	60	24.2	16.6	1.8	45.4	36.2
		90	25.7	19.5	1.6	41.8	37.1

Table S7. Results of the catalytic tests for the $V_{6.0}$FAUdes sample.

SAMPLE	T_r [°C]	Flow [ml/min]	Conv [%]	S_{C3H6} [%]	S_{C2H4} [%]	S_{CO2} [%]	S_{CO} [%]
$V_{6.0}$FAUdes 0.5ml/0.5ml quartz/catalyst	400	30	2.6	45.1	0	49.6	6.1
		60	4.2	60.1	0	31.8	8.1
		90	9.5	63.6	0	22.3	14.2
	450	30	6.1	46	0.1	40.8	13.1
		60	4.4	51.6	0.2	35.9	12.4
		90	8.9	65	0.3	19.0	15.8
	500	30	20.2	31.9	0.2	46.3	21.7
		60	11.9	43.5	0.2	39.1	17.3
		90	8.7	56.8	0.3	27.1	15.9

Table S8. Comparison of the initial catalytic performance of the studied samples with their activity after 40h (T = 450 °C, flow 60 ml/min).

SAMPLE		Conv [%]	S_{C3H6} [%]	S_{C2H4} [%]	S_{CO2} [%]	S_{CO} [%]
$V_{1.0}$FAU	1st run	7.7	33.7	1.0	42.9	22.5
	after 40h	7.5	28.5	0.9	42.8	27.7
$V_{3.0}$FAU	1st run	13.0	29.9	0.6	43.3	26.1
	after 40h	8.1	3.2	0.6	43.0	26.2
$V_{6.0}$FAU	1st run	14.7	19.6	0.4	54.8	23.2
	after 40h	53.4	4.9	3.5	65.8	25.7
$V_{1.0}$FAUdes	1st run	3.7	24.8	0.6	57.4	17.2
	after 40h	4.6	20.0	0.5	63.4	15.8
$V_{3.0}$FAUdes	1st run	5.7	26.7	0.6	41.6	31.1
	after 40h	17.1	35.3	0.5	38.9	25.3
$V_{6.0}$FAUdes	1st run	4.4	51.6	0.2	35.9	12.4
	after 40h	6.0	47.7	0.1	42.3	9.9

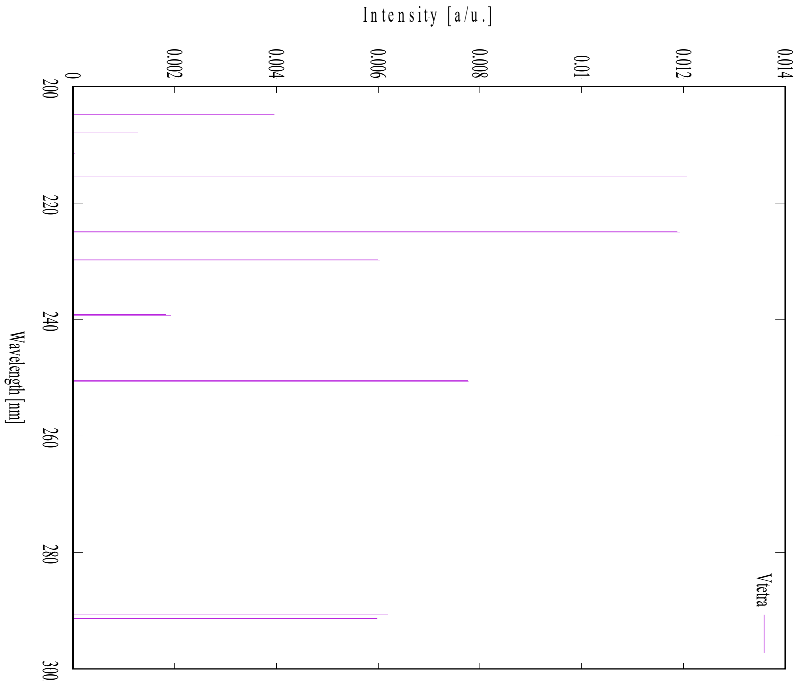

Figure S1. Theoretical (TD-DFT: PBE/def2-TZVP) UV-VIS spectrum of the $VO(OH)_3$ complex, in which vanadium is located in the tetrahedral environment.

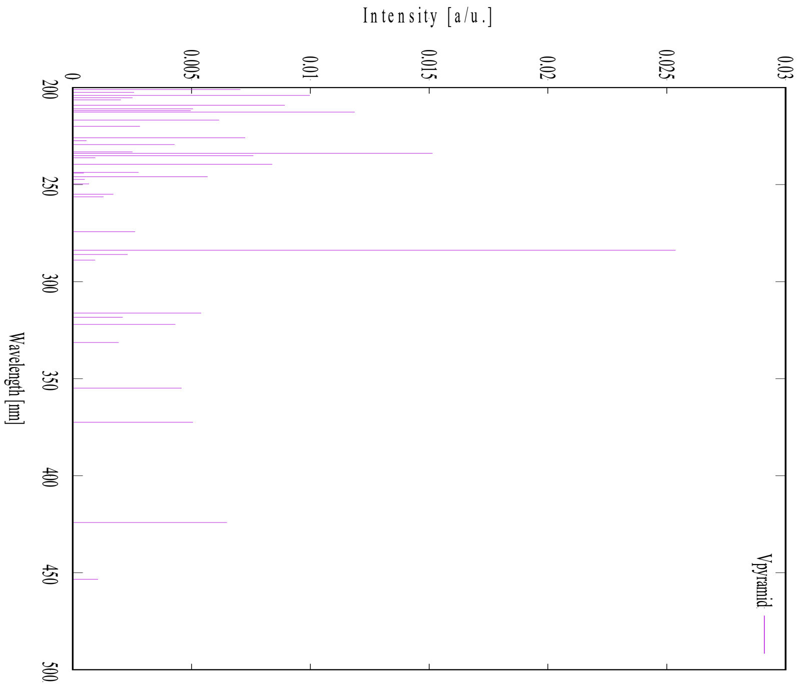

Figure S2. Theoretical (TD-DFT: PBE/def2-TZVP) UV-VIS spectrum of the $VO(OH)_4^-$ complex, in which vanadium is located in the square pyramid environment.

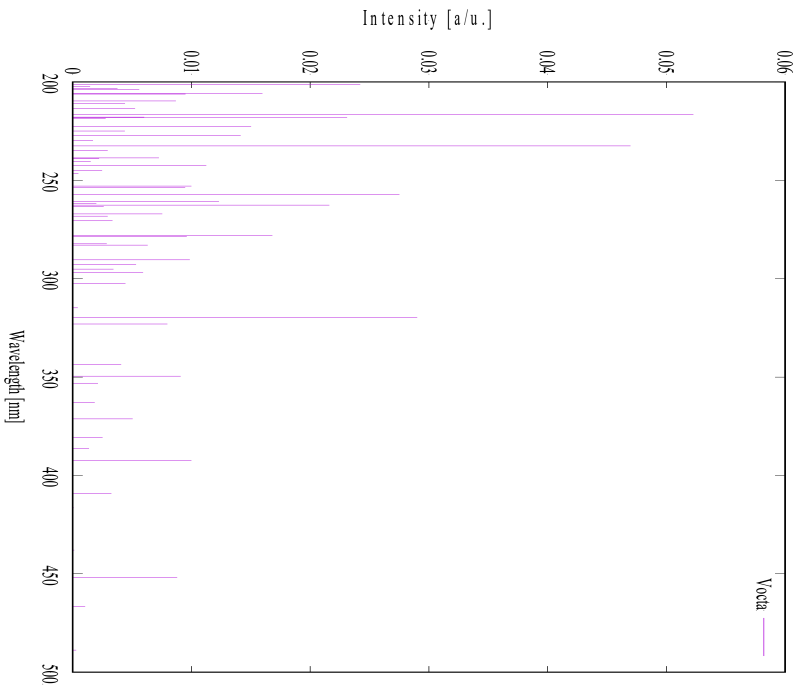

Figure S3. Theoretical (TD-DFT: PBE/def2-TZVP) UV-VIS spectrum of the $VO(OH)_5^{2-}$ complex, in which vanadium is located in the octahedral environment.

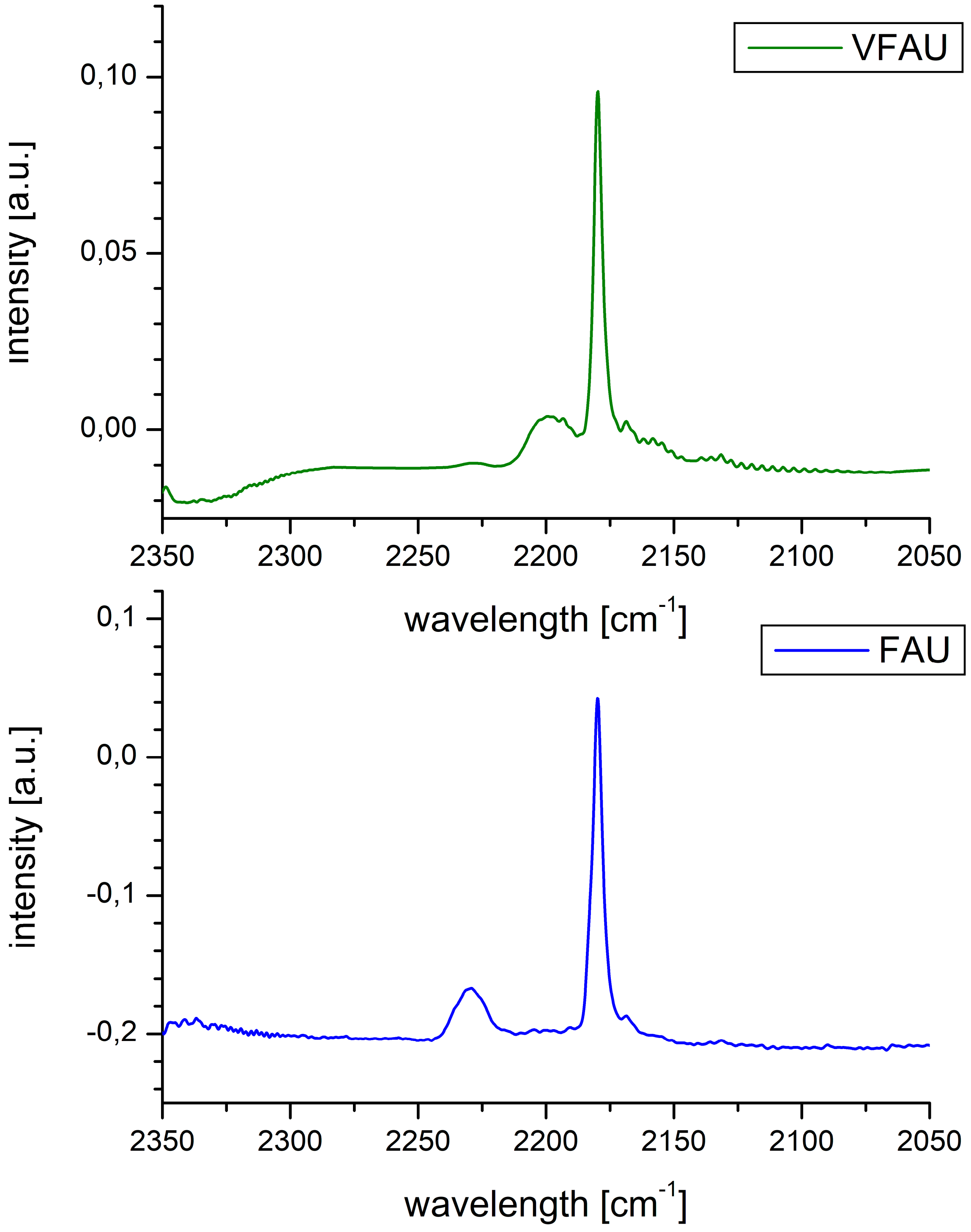

Figure S4. Exemplary IR spectra of the studied samples.

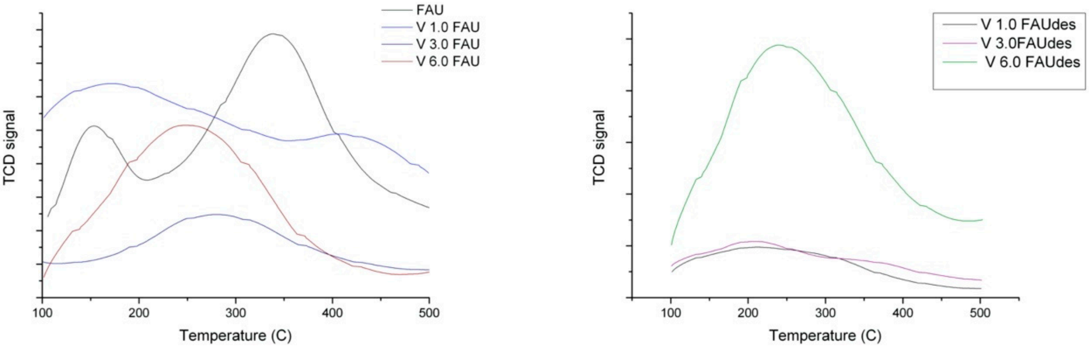

(a) (b)

Figure S5. NH_3-TPD profiles for the VxFAU (a) and VxFAUdes (b) series.
